# Gendered affective representations in AI-assisted music videos: a qualitative multimethod study

**DOI:** 10.3389/fpsyg.2026.1979653

**Published:** 2026-10-21

**Authors:** Yaşar Kızıltan, Ayhan Dolunay, Savaş Güngör

**Affiliations:** 1 Department of Visual Communication Design, Faculty of Communication, Near East University, Nicosia, Cyprus; 2 Department of New Media and Communication, Faculty of Communication, Near East University, Nicosia, Cyprus

**Keywords:** artificial intelligence, gender, music video, representation of emotion, synthetic visual culture

## Abstract

**Introduction:**

Generative artificial intelligence (AI) has become increasingly visible in music-video production, influencing both production practices and the ways in which emotion, bodies, and gender are portrayed visually. This study examines gendered emotional representation in AI-assisted music videos.

**Methods:**

A qualitative multimethod design combined interpretive visual analysis of four purposively selected AI-assisted music videos with asynchronous written expert interviews involving six creative-industry professionals and four psychology experts (*N* = 10). The videos were analysed through an interpretive coding framework covering emotion, gender, body and face, power relations, AI aesthetics, and traces of bias.

**Results:**

Across the cases, emotion was conveyed not only through facial expression but also through atmosphere, colour, movement, editing, and spatial transitions. The selected works sometimes reproduced familiar conventions, including heteronormative intimacy, militarised masculinity, and idealised female bodies, but also unsettled them through female anger and solidarity and mutable synthetic bodies. Ambiguity in a body’s appearance did not necessarily unsettle its gendered narrative role. Industry accounts described how prompting, selection, revision, and post-production shape representations, while general psychology-expert perspectives informed their interpretation.

**Discussion:**

The findings position AI-assisted music videos as co-produced sites of synthetic visual culture in which established gendered conventions may be repeated, negotiated, or challenged. Neither expert group provided direct evidence about the production or reception of the selected videos. The findings underline the practical importance of representational review, consent, diversity, and transparent disclosure in AI-assisted cultural production.

## Introduction

Artificial intelligence has moved from a specialist research field into everyday communication and cultural production. Its history is commonly traced from Turing’s question of machine intelligence (Turing, 1950) to the 1956 Dartmouth workshop, which helped establish AI as a distinct field (Muthukrishnan et al., 2020). Since the early 2020s, generative systems have expanded the capacity to produce text, images, sound, and moving images, bringing AI more directly into creative workflows.

Music and communication are among the fields in which the use of artificial intelligence has recently become more visible. Music videos have evolved beyond a means by which artists bring their songs to listeners; they have become a form of expression that combines visual and auditory aesthetics and directly represents the artist’s image and communication. The role and use of generative AI in these music videos are likewise increasing steadily. As in all works of visual culture, generative AI systems offer new experiences and possibilities to artists, creative teams, and audiences alike in music videos. These developments have opened up possibilities for individual creators as well as the wider sector. In visual culture, however, AI now does more than offer technical efficiencies or reduce production time and cost. It can also affect the way a work tells its story and frames gender, emotion, and the emotional basis of the narrative. The gains in speed, efficiency, and customisation therefore sit alongside continuing concerns about privacy, bias, fairness, and changes to human labour (Dolunay, 2024).

Once understood mainly as visual accompaniments to songs and, particularly from the 1980s onward, as promotional devices for the popular-music industry, music videos now fulfil a much wider role. What matters centrally is what emerges from the relationship between image and sound. Lighting, bodies, movement, setting, imagery, and editing work as communicative resources; together with the song, they shape emotional codes and representations of gender.

Recurring arrangements of bodies, gazes, movement, costume, and space also assign gendered roles within music videos. Meaning arises from the interaction of sound and image, not from lyrics or visuals considered in isolation (Negus, 1996; Railton and Watson, 2011). Music videos can therefore be read as cultural texts that reveal how particular emotions become attached to certain bodies and forms of agency.

AI-assisted production adds questions of responsibility and the right to representation to this relationship. Choices about how bodies and emotions appear on screen have ethical implications as well as aesthetic and technical ones, locating this work within a socio-technical process (Kambur and Dolunay, 2024).

The present study explores the gender representations and emotional codes found in music videos made with generative-AI support. It does not approach the model as an independent author; instead, the completed video is understood as the combined result of model behaviour, prompting, human selection, and post-production.

The central problem of the study may be formulated through the following question: “Which emotions do AI-assisted music videos represent through which visual codes, and with which gender patterns are these representations associated?” This question demonstrates that the use of generative AI outputs in the creative industries merits consideration not only in terms of the innovations and technical possibilities it introduces, but also within the fields of psychology, sociology, visual culture, ethics, and gender studies.

### Research questions

1. Which emotions are represented through which visual codes in music videos produced with the support of generative AI systems?
2. How are these representations associated with particular gender codes?
3. How do the selected AI-assisted music videos reproduce, negotiate, or challenge established gendered conventions of emotion?
4. How do industry professionals assess the role of AI and human creative decisions in representations of gender and emotion?
5. How can psychology experts’ general perspectives on gender and emotional representation inform the interpretation of AI-assisted music videos?

### Contribution of the study

Research has examined AI-generated visuals, music videos, gender bias, and emotional representation from several perspectives, but few studies have considered these issues together in AI-assisted music videos. This intersection provides the point of departure for the present study. Its contribution lies in bringing production practice, visual analysis, and general psychology-expert perspectives into the same qualitative framework.

This study contributes to the literature at several levels. First, by conceptualising AI-assisted music videos in relation to synthetic visual culture and representations of emotion and gender, it situates debates on generative AI in this field within a theoretical framework. Second, it develops an approach that considers representations of emotion and gender together within synthetic visual culture. Third, the interviews with industry representatives provide insight on production workflows, prompt entry, and pre- and post-production processes. Fourth, with the contribution of psychology experts, the study offers an analytical perspective in areas such as representations of emotion, perceptions of the body and face, awareness of signs, and the construction of synthetic visual culture, while offering ethical and practical implications.

More directly, the study contributes to emotion science by asking how emotional meaning is made perceptible in synthetic audiovisual environments. It examines the face alongside posture, movement, colour, space, and editing, with music and lyrics providing context, and considers how these cues are reorganised when generation or digital alteration enters the production of bodies and settings. The psychology experts were not asked to assess the selected videos. Their general written responses connect the visual analysis to debates over emotion recognition, context, and interpretation, without standing in for audience data. The contribution concerns the arrangement of emotional cues in generative media: which emotions become legible through particular gender conventions and synthetic forms. It makes no claim about what viewers actually feel.

### Theoretical background

#### The age of digital communication

Teichert (2019) describes digital transformation as an ongoing process. In communication, the shift towards networked platforms has made interaction faster, more accessible, and able to reach further. Concerns about privacy, information disorder, and ethics have grown alongside these changes (Çaycı and Çaycı, 2017). Generative AI belongs to this broader shift, since digital communication now encompasses the automated and collaborative creation of content as well as its distribution.

#### Generative artificial intelligence and synthetic visual culture

Generative AI systems create content, including text, images, sound, music, video, and animation, by drawing on patterns learned from training data. Work on visual generation includes evaluations of Generative Adversarial Networks (Çelik and Talu, 2020), alongside subsequent developments in diffusion and related architectures. These technical distinctions are not the main issue here. The relevant question is how the resulting material is taken up within a human creative process.

The model is only one influence on that process. What reaches the finished work also depends on model design and training material, as well as the wording of prompts, the use of reference images, decisions about which outputs to keep, subsequent revision, and post-production. Generative production is thus better regarded as an arrangement between people and machines than as an autonomous creative act.

Questions of representation become particularly important from this socio-technical standpoint. Even an output that appears visually convincing can reproduce familiar assumptions about gender, emotion, beauty, authority, or vulnerability. At the same time, creators exercise judgement by retaining, discarding, and modifying what the system produces.

Generative systems give artists and designers further ways to experiment with images. Variations can be produced quickly, including environments, mutable bodies, and transitions whose conventional production would entail different constraints and resources. These possibilities change where creative choices are made in the production process. They do not remove human authorship or the responsibilities attached to it (Manovich, 2019).

Synthetic visual culture refers here to images that carry visual and emotional force even though they do not record events in the physical world. A text-to-video system can produce apparent camera movements, transitions, faces, and bodily proportions without a location shoot. If viewers find these images convincing, their interpretation may depend less on whether the material was filmed or generated (Baudrillard, 2010; Manovich, 2019; Westerlund, 2019).

For Gross and Petrean (2025), “synthetic visual culture” encompasses the production and circulation of generative-AI imagery and the practices through which such imagery acquires meaning. In this setting, visual production no longer rests solely on the artist’s technical skill. It emerges through the interaction of datasets, algorithmic models, textual prompts, and human choices. AI-generated music videos constitute a prominent example of synthetic visual culture because they visualise the emotional and narrative content of music through generated or digitally altered bodies, spaces, characters, and aesthetic forms, sometimes combined with camera-recorded performance. Music videos thereby cease to function merely as visual accompaniments to songs and instead become cultural spaces in which reality, identity, the body, gender, and modes of emotional representation are reproduced through artificial intelligence (An and Lee, 2025).

Products of visual culture created with the support of generative AI systems are generally not treated as representations of reality, but as new visual worlds that have been artificially designed and constructed. Music videos also belong to this category. AI-generated music videos may therefore be directly associated with Baudrillard’s theory of simulation. Viewers can respond emotionally to spaces, bodies, events, and people that do not in fact exist. Although such images are artificial and have no real existence within synthetic visual culture, the responses and emotions they elicit, and the reactions to the representations they produce, may still carry socially and psychologically meaningful consequences (İpek and Ermiş İpek, 2025).

#### Music videos and artificial intelligence

Combining a recorded song with moving images, the music video has long provided a means of artistic expression as well as promotion. Its development through music television and, later, online platforms gave rise to an audiovisual language with conventions extending beyond the illustration of song lyrics (Çelikcan, 1996; Vernallis, 2004).

Performance, camera perspective, colour, movement, rhythm, and editing all contribute to how a music video represents bodies, identities, desire, and emotion (Railton and Watson, 2011). Studying these works allows us to ask where image and sound reinforce a meaning and where they pull it in different directions.

Following the establishment of MTV (Music Television) in the United States in 1981, music videos began to reach broader audiences. Its first music video, broadcast on 1 August 1981, was the Buggles’ “Video Killed the Radio Star” (Guinness World Records, n.d.). Constructed promotional videos were therefore part of its programming from the outset. While MTV and its music videos became an effective means of promoting and selling an album, it also created stars and marketed not only music but products; at times, programme content was filled entirely with advertising elements. MTV had a major impact on increasing the album sales of the new musicians it featured (Erşanlı, 2016). This, in turn, encouraged artists to make additional efforts to have their music videos broadcast on MTV and to produce videos capable of attracting attention.

In the early 1980s, when MTV was only beginning to become influential, music videos were supplied to television channels free of charge. This arrangement offered advantages to both parties. Because the television industry struggled to find programmes to broadcast, music videos provided both a cost-free means of filling uninterrupted airtime and a way of drawing young people to the screen. The music industry, in turn, gained a medium through which it could readily promote songs and thereby increase sales. Viewers drawn to the screen encountered intensive product advertising directed at them, because the purpose of repeatedly broadcasting music videos was to familiarise audiences with the song or singer and encourage them to purchase the product (Ersöz, 2002).

With the spread of internet access, digital cameras, editing software, and online video platforms in the 2000s, combining and distributing audiovisual material became easier. YouTube and later social-media platforms shifted music-video circulation away from scheduled television and enabled immediate global sharing, commentary, and recirculation (Erşanlı, 2016).

Although music is an auditory art form, it should not be forgotten that it is also part of a profit-oriented consumer culture and therefore requires advertising and, by extension, the media. Music videos take the music produced beyond a purely auditory experience, give it a new perspective by combining the visual and the auditory, and sometimes reinforce the meaning of the song’s story or intended message, while helping the work gain greater media exposure and reach wider audiences. In the sector’s own terminology, they serve as a “promotional instrument” that strengthens album sales and chart performance. Accordingly, when discussing a song that has a music video, the art created simultaneously in visual and auditory form must be considered as a whole (Erşanlı, 2012). At the same time, music videos are more than promotional instruments that visually package popular music; they are cultural texts in which representations of identity, body, and emotion are produced. The rhythm and lyrics of the song, together with the manner of performance, work through camera movement, editing, colour, space, and bodily aesthetics to build an emotionally intense viewing experience. The music video should therefore be considered a distinctive media form in which visual, auditory, and verbal codes operate together.

Given the importance of visuality to music, generative AI has also begun to be used in music videos and visuals. Recent advances in digital technologies have substantially increased the capacity to generate highly realistic images and videos through computer graphics and AI algorithms (Westerlund, 2019). As the possibilities for using AI in video-content production have expanded, it has become possible to employ AI technologies at every stage of film production, including pre-production, production, post-production, distribution, and exhibition (Türten, 2024). These capabilities are not limited to film: AI has begun to be used across visual domains such as social-media content, advertising videos, and music videos, and its role in these sectors is still developing.

#### Affective artificial intelligence, emotional representation, and visual meaning

Emotion research includes category-based approaches to basic emotions (Ekman, 1992) and dimensional accounts organised around valence and arousal (Russell, 1980). Constructionist work further emphasises that emotional experience and recognition are context-sensitive (Barrett, 2017). In audiovisual media, emotion is therefore not reducible to facial expression; it is also organised through bodily posture, colour, light, space, rhythm, sound, and editing.

Work on affective AI has examined how digital systems detect, classify, predict, and represent human emotion (McStay, 2018). A persistent problem is the tendency to treat emotions as fixed, universal categories despite the cultural and contextual variation in how they are expressed and interpreted. The present analysis makes no claim that AI can disclose the inner states of either viewers or characters. The object of analysis is the emotional meaning of the screen representation.

We therefore read emotional representation in the selected videos through culturally shaped visual codes. Psychological theory helps interpret those codes, but the images cannot serve as direct evidence of ‘real emotion.’ Nor can this analysis establish viewers’ internal responses, which were not measured.

Empirical studies help explain why the distinction matters. Lecker et al. (2025) found that bodily and contextual information can be more diagnostic of intense fear in real-life situations than facial information alone. Findings on synthetic faces raise a different issue. White AI-generated faces have been mistaken for human faces more often than actual White human faces, while generated emotional expressions have been classified more accurately and rated as more intense than posed photographs (Miller et al., 2023; Kramer et al., 2026). Realism and emotional equivalence cannot, then, be assumed to coincide. Diekhof et al. (2025) also report measurable physiological responses to small departures from human appearance in immersive environments, placing virtual agents within the uncanny valley. These studies give reason to examine bodily and contextual cues alongside the realism and synthetic form of an image, rather than relying on the face alone.

#### Gender, algorithmic bias, and representation

In this study, gender concerns the roles, behaviours, and meanings socially attached to perceived differences between women and men. It is understood as a cultural construction, extending beyond biological differences. Hall’s (1997) account of representation places a similar emphasis on meaning: language and visual signs produce meanings through which reality is understood. Portrayals of women and men in the media are therefore part of how gender roles are made and circulated.

Butler (1990) locates gender in the repetition of bodily acts, behaviours, and discourse. In this account, gender is performative rather than a fixed identity. Visual media contribute to that repetition through the postures, clothes, expressions, and roles assigned to characters. Viewers encounter recognisable conventions of femininity and masculinity, for example when women appear vulnerable, passive, or seductive and men are presented as powerful, aggressive, and emotionally restrained.

Mulvey’s (1975) “male gaze” describes a familiar division in visual representation: a male character acts and looks, and a female character is presented for his—and the spectator’s—visual pleasure. Close-ups of selected body parts and the choice of camera angle can sustain this division. Although a generated image need not involve a physical camera, its framing, poses, and bodily proportions may retain these conventions of objectification. The association of male characters with power, aggression, authority, and emotional control may, in turn, be evaluated within the framework of Connell and Messerschmidt’s (2005) concept of hegemonic masculinity.

Research on music videos likewise shows that gender may be represented unequally. Wallis (2011) found that women in music videos were associated with behaviours involving sexual connotations, vulnerability, and subordination more often than men, whereas men were associated with greater aggression. Aubrey and Frisby (2011) similarly found that female artists were subjected to sexual objectification more intensely than male artists and were represented according to stricter standards of physical appearance. These findings show that music videos are not merely products that promote songs, but cultural texts in which social meanings relating to the body, sexuality, power, and emotion are produced.

With the introduction of generative AI into visual-content production, gender representation has also become a question of algorithmic bias. Bias in computer systems may arise from pre-existing social inequalities, technical design choices, and the contexts in which systems are used (Friedman and Nissenbaum, 1996). Models trained on large collections of visual and textual material can therefore recirculate patterns already present in media culture.

Naik and Nushi (2023) showed that the text-to-image models DALL·E 2 and Stable Diffusion could generate stereotyped images concerning occupations and personality traits even in response to gender-neutral prompts. Sun et al. (2024), meanwhile, found that women were underrepresented in images of male-dominated occupations generated by DALL·E 2 and were depicted with smiling facial expressions more frequently than men. These findings demonstrate that algorithmic bias affects not only the numerical visibility of women and men, but also the bodily and emotional attributes assigned to them.

Body representation is similarly consequential. In an experiment with young women, exposure to idealised, fit-looking gen-AI images produced poorer body-image outcomes than exposure to body-diverse, functionality-focused imagery; disclosure that the images were AI-generated did not remove these effects (Alleva et al., 2026). The significance of a synthetic body therefore depends not simply on whether it is artificial, but on the bodily ideal it reproduces and the conditions in which viewers encounter it.

The gender representations visible in AI-assisted music videos should not be attributed to an algorithm alone. The model, prompt wording, reference material, selection of outputs, editing, and post-production all contribute to the final result. The videos are therefore approached here as sites of cultural representation in which gender norms may be reproduced, negotiated, or questioned through a co-produced process.

## Method

### Research design

This study uses a qualitative multimethod design combining interpretive visual analysis with asynchronous written expert interviews based on open-ended question schedules. Interpretive analysis makes it possible to examine not only the elements visible in an audiovisual text but also the cultural codes and relations of meaning established among them (Yanow and Schwartz-Shea, 2006). Because music videos bring together mise-en-scène, body, face, colour, light, movement, editing, and rhythm, this approach was considered appropriate for the research questions (Weber, 1990).

The methods address related but distinct questions. Visual analysis examines the representations in the four completed videos. Industry responses concern professional practices that may shape such representations, while psychology-expert responses address general questions about emotion, bodies, and synthetic imagery. Integration takes place through comparison within the shared analytical dimensions and in the interpretation of the findings. We use these connections to develop and qualify interpretations. Agreement on a theme does not independently verify a scene reading or an account of production.

The visual analysis used interpretive coding rather than counts. Categories drawn from theory were considered alongside open codes developed from the videos, following the iterative movement between coding and interpretation described by Braun and Clarke (2006). Comparison across the four cases was organised around six dimensions: emotion, gender, body and face, power relations, AI aesthetics, and traces of bias.

Each participant received an open-ended question schedule by email and replied individually in writing. Separate schedules were used for industry professionals and psychology experts. Within each group, common topics enabled comparison, while the open format allowed participants to explain their views in their own terms (Patton, 2018; Roberts, 2020). There was one asynchronous exchange, with no live interview, group discussion, or follow-up questions. Approval was obtained from the Near East University Scientific Research Ethics Committee, and every participant gave written informed consent.

### Population, sample, and case selection

The population of interest was music videos made with AI assistance. We selected four cases purposively, seeking variation in production methods and visual treatment. This follows the rationale for choosing information-rich cases relevant to the research questions (Patton, 2018; Yağar and Dökme, 2018). The sample covers text-to-video generation, sequences of generated stills, filmed performance combined with AI-assisted alteration, and AI-assisted animation. Keeping the sample to four videos allowed detailed reading of each work and comparison of emotional and gendered representation across these different processes. It provides no basis for estimating how common the resulting patterns are in music videos generally. Initial descriptions of emotional atmosphere and gender cues helped guide the selection; these remained provisional and did not determine the findings or fix the analytical categories. The following criteria guided selection:

1. The video must have been generated by artificial intelligence or must have made conspicuous use of AI-assisted visual-generation tools during the production process.
2. The video must contain figurative representations involving humans, humanoids, avatars, bodies, faces, or gender codes.
3. The video must contain a clearly discernible emotional atmosphere.
4. The sample was structured to include four preliminary contrasts in emotional atmosphere—nostalgia/loss/vulnerability; anger/threat/fear; desire/female anger/agency; and surveillance anxiety/technological uncanniness/apparent bodily ambiguity—solely to maximise variation among the cases.
5. The sample must enable the comparison of different production tools and aesthetic strategies.
6. The sample must allow representations of femininity, masculinity, and apparent gender ambiguity or bodily fluidity to be discussed together.

The selected cases are summarised in Table 1.

**Table 1:** Overview of selected cases and preliminary sampling contrasts. These descriptors guided variation in selection; they are not final analytical findings.

Case	Video	AI tool/process	Preliminary emotional contrast for sampling	Preliminary gender-related sampling contrast
1	Washed Out – The Hardest Part	Sora / text-to-video generation	Nostalgia, loss, vulnerability	Female–male romantic couple; intimacy and memory
2	Disturbed – Bad Man	Midjourney images / sequential visual flow	Anger, threat, fear	Male leader and soldier figures; authority and violence
3	Björk ft. Rosalía – Oral	Filmed performance combined with AI-assisted likeness alteration and visual effects	Desire, female anger, agency	Female performers’ likenesses; combat, bodily agency, and idealised appearance
4	Peter Gabriel – Panopticom	Stability AI / AI-assisted animation	Surveillance anxiety, uncanniness, alienation	Non-human/cyborg forms; apparent bodily ambiguity

Formal emotion coding began only after the cases had been selected. The preliminary descriptions could be confirmed, revised, divided, or extended during analysis, and the categories reported in the findings were developed through the three-round coding procedure described in Analytical coding framework.

A distinction between an initial contrast and a final interpretation is particularly important for Panopticom. Its non-human bodies initially made it a useful contrast with the more conventionally gendered figures in the other cases. Closer consideration of bodily appearance alongside narrative roles qualifies that contrast: visually unstable bodies coexist with a male–female story of desire and sacrifice. Preliminary ambiguity therefore does not function as a predetermined finding of gender neutrality.

Participants were selected purposively for knowledge and experience relevant to the research questions (Palinkas et al., 2015; Başkale, 2016). Eligibility required at least 5 years of professional experience in the participant’s general field, together with relevant work in AI-assisted visual production for industry participants or work on the study’s psychological concerns for psychology experts. The experience threshold concerned professional practice, not 5 years of work with recent generative-AI tools. Eight industry professionals and six psychology experts were invited by email; six and four, respectively, returned responses. All 10 respondents were included. The achieved sample therefore reflects responses from an eligible, purposively approached group rather than statistical representativeness or a claim of demonstrated saturation.

Industry participants were selected from AI-assisted visual production in Türkiye and had not worked on the four selected videos. This avoided relying on creators’ evaluations of their own productions, but also means that their accounts cannot establish how those particular videos were made. Psychology experts were selected for relevant work on emotion, gender, media, or human–computer interaction. Across both groups, the rationale was to obtain informed perspectives on the analytical questions; professional experience does not remove interpretive commitments or make an account inherently objective.

### Data collection

For each case in the video analysis, consideration was given to the video in its entirety, selected key frames, song lyrics, statements by the director or producer where available, behind-the-scenes content, press releases, and open-source information on the use of artificial intelligence. These sources informed our understanding of production and the reading of the images. The analysis itself focused on visual representation. Music and lyrics supplied context for that reading; references to rhythm, vocals, or lyrical themes do not amount to a separately coded corpus or a systematic musicological analysis. Production documents were likewise used as contextual evidence and distinguished from what could be observed in the videos.

The industry question schedule covered AI’s role in production; the description of emotion and gender in prompts; participants’ reasons for using these tools; encounters with stereotyped outputs; selection and revision; representational responsibility; and expectations for future practice. Participants could describe experiences and explain their reasoning without choosing from predetermined response options. The questions concerned participants’ own professional practice, not the production histories of the four videos.

Data collection ran from 1 June to 1 July 2026. An email invitation explained the study and included an informed-consent form. After giving written consent, participants received the questions and returned their answers by email in a single round. They chose whether to respond in Turkish or English. We analysed these written answers directly, so there were no audio or video recordings to transcribe. The authors translated the Turkish responses into English. Quotations retain either the participant’s original English wording or the authors’ translation of the Turkish response; they are not paraphrased accounts.

As participants answered asynchronously, there was no interview session whose duration could be reported in minutes. We set no fixed response length and did not calculate a mean word count. The returned answers were judged sufficient for the exploratory questions, and no further questions were sent. Writing allowed participants time to formulate an account, although a single exchange offered fewer opportunities to clarify brief or ambiguous answers than repeated questioning would have provided.

Questions for the psychology experts concerned emotional cues in synthetic images, gendered bodies and faces, idealisation and body image, trust, uncanniness, and the ethics of avatars and deepfakes. We did not show them the videos or provide extracts, summaries, or preliminary interpretations. Their answers therefore address the subject generally. The authors related these views to the visual findings during interpretation. This avoided eliciting responses to our own case readings, but it also means that the answers cannot independently confirm those readings or establish audience responses.

### Analytical coding framework

We coded the material jointly, working with both theory-led themes and open codes arising from the material. The literature and research questions guided our attention without setting an exhaustive list of categories in advance. This left room to examine patterns specific to the videos. The process drew on Braun and Clarke’s (2006) account of revisiting data and codes as broader interpretations develop; the authors did not carry out separate independent coding.

The visual reading moves between frames, sequences, and the complete narrative. Individual frames provide evidence of features such as a face, posture, costume, or setting; scenes and sequences show how those features develop through movement and editing; the whole-video narrative supplies the context for interpreting relationships, agency, and change. The examples reported below use the scene or sequence as the principal explanatory unit and return to frames and the wider narrative where needed. These levels are not treated as independent observations or frequency counts. Written responses are interpreted through meaning-bearing passages, with each passage read in the context of the respondent’s full account.

The coding process was organised in three iterative rounds. In the first round, the video material was reviewed closely and open codes were assigned to recurrent visual and affective features, including expressions, bodily positions, settings, colour, movement, gender cues, power relations, and visible characteristics of AI-generated imagery. Written responses were also read and coded in relation to the same broad concerns, with the source of each interpretation retained. In the second round, these open codes were compared with the theory-led categories developed from the literature and the research questions. Codes that described closely related phenomena were merged or grouped, while materially distinct codes were retained as subcodes. In the third round, the authors reviewed the provisional structure across all four cases and the interview material, refining labels and boundaries and checking whether the resulting categories remained internally coherent while still distinguishing analytically different patterns.

Open codes were therefore not mechanically forced into predetermined categories. The open codes sometimes made a broad category more precise and sometimes required us to qualify or extend it. Theory, in turn, helped relate individual observations to larger patterns. The final framework retained specific codes within six dimensions: emotion, gender code, representation of body and face, power relation, AI aesthetic, and trace of bias.

The authors differed on relatively few readings. When this occurred, we returned to the relevant scene, segment, or written response and discussed it until we reached a shared interpretation. Because the coding was developed together, no independent-coder reliability assessment or numerical agreement coefficient was used. Agreement documents the reading reached by this research team; another team might interpret the material differently. We also revisited how our disciplinary background and knowledge of the literature directed attention towards particular features. Returning to the sources helped us separate what was depicted or stated from what we inferred through theory (Braun and Clarke, 2006; Yanow and Schwartz-Shea, 2006).

To bring the methods together, we read passages from the written responses in relation to particular visual interpretations, asking what each source could contribute. PE3’s discussion of bodily and contextual cues, for instance, helps interpret the spatial transitions in The Hardest Part. IP1’s description of output selection places a limit on attributing an idealised appearance directly to a model. A general expert statement can illuminate the basis or limits of a visual reading without proving it. Worked examples in the findings make these relationships explicit, including points at which an initially broad interpretation needs qualification.

The analytical dimensions and guiding questions are summarised in Table 2. Table 3 traces selected observations through codes to interpretations for The Hardest Part, Oral, and Panopticom, distinguishing visual evidence from production accounts and general interview perspectives.

**Table 2:** Analytical coding framework.

Dimension of analysis	Examples of codes	Analytical question
Emotion	Love, grief, nostalgia, fear, anger, desire, threat, vulnerability	Through which visual signifiers is each emotion constructed?
Gender code	Femininity, masculinity, androgyny, gender ambiguity	With which gender codes is the emotion associated?
Representation of body and face	Beauty, vulnerability, strength, age, idealisation, deformation	How is the body used as a bearer of emotional meaning?
Power relation	Authority, violence, control, gaze, passivity, agency	Who looks, who is looked at, and who is constructed as the acting subject?
AI aesthetic	Hyperreality, hallucinatory imagery, facial/bodily inconsistency, synthetic atmosphere	How does the visual structure specific to AI affect emotion?
Trace of bias	Production of stereotypes, lack of diversity, normative beauty, militarised masculinity	Does the representation reproduce existing social patterns?

**Table 3:** Worked examples linking observations, codes, and interpretations.

Case and evidence	Observation or contextual evidence	Codes	Interpretation and qualification
The Hardest Part Visual sequences and narrative context	School encounters, car journeys, and later-life scenes repeatedly return to the same female–male relationship. Spatial transitions connect these episodes through memory and loss.	Romantic intimacy; nostalgia; vulnerability; couple-centred narrative.	The relationship provides the recurring basis for the account of emotional life. This supports a limited reading of heteronormative intimacy; the mere presence of a female–male couple would not be sufficient. PE3’s general comments help explain the attention to setting and transitions, without confirming this particular interpretation.
Oral Visual performance and production context	Figures bearing the artists’ likenesses wield swords, perform defensive movements, fall, and rise in a white setting. Their polished appearance coexists with physical action. The production account identifies them as allies (Pitchfork, 2023).	Bodily agency; controlled aggression; aestheticisation; solidarity informed by the production account.	Physical action supports the code of agency, and polished appearance supports aestheticisation. The stronger reading of eroticisation would require evidence of sexualised framing. IP1’s description of selection and revision also limits an explanation based on AI alone, given the contributions of filmed performance, direction, and post-production.
Panopticom Visual transformation and the director’s account	Human features give way to mechanical surfaces and shifting bodily forms. Separately, the director describes a male protagonist who develops feelings for a female cyborg and gives her his heart (Gabriel, 2023b).	Bodily ambiguity; transformation; gendered desire; sacrifice.	Ambiguity is retained as a code for bodily appearance only. The narrative account retains male and female roles in desire and sacrifice, qualifying a reading of gender neutrality. PE2’s general comments on uncertainty in non-human expressions inform interpretation of appearance, without determining the characters’ gender.

The examples illustrate the links between observations, codes, and interpretations; they are not frequency counts or independent observations. Connections to interview material were made by the authors using general expert responses. Production accounts are distinguished from visual descriptions.

### Interviews

The interview component has two complementary layers: accounts from industry representatives involved in creative production and general perspectives from psychology experts. Participant identities were anonymised. Industry representatives are identified as IP1–IP6 and psychology experts as PE1–PE4 throughout the manuscript.

#### Industry representatives

The six industry representatives each had at least 5 years of professional experience and collectively worked across direction, creative development, music-video production, post-production/VFX, AI visual generation, and art or artist representation. This range was selected so that decisions made during idea development, prompt writing, visual generation, selection and filtering, post-production, and publication could be considered together. Qualitative studies of communication professionals’ views on AI indicate that interviews of this kind can help identify the practical advantages, limitations, and ethical questions associated with its use (Dolunay, 2024).

The industry interviews focus particularly on the following themes: the role of AI in the production process; descriptions of emotion and gender in prompt writing; recurring patterns of bodies, faces, and emotions in AI outputs; experiences of encountering biased or problematic outputs; representational responsibility; creative control; and the relationship between human labour and algorithmic production. These general professional accounts are compared with the visual themes by the authors; they are not testimony about participation in the selected productions.

#### Psychology experts

Four psychology experts (PE1–PE4) with relevant knowledge in areas including emotion, social or gender psychology, media psychology, cyberpsychology, and human–computer interaction were included. Each had at least 5 years of professional experience and relevant work on the study’s concerns. Their general responses informed the study’s psychological discussion without being treated as case assessments or a substitute for audience research.

The psychology schedule addressed the following general topics, without presenting individual videos or the authors’ interpretations: the visual cues through which emotions may be recognised in AI-assisted imagery; how emotions such as nostalgia, anger, threat, desire, and uncanniness are constructed through the body, face, colour, space, and rhythm; how female, male, avatar, or cyborg bodies affect audience perception; the relationship between idealised synthetic images of faces and bodies and norms of beauty; and how avatar/deepfake aesthetics affect feelings of trust, discomfort, identification, or distance. These topics describe the scope of the schedule. The resulting views provide a complementary interpretive perspective rather than validation of case readings or audience effects.

### Findings

#### Synthetic nostalgia and gendered intimacy: washed out – the hardest part

The music video for Washed Out’s ‘The Hardest Part’, directed by Paul Trillo, was presented as the first official music video made with OpenAI’s Sora. Its text-to-video workflow supports long, dream-like transitions that would involve different production resources and constraints in a conventional live-action or visual-effects process. Within a narrative that continually moves forward through time, the video follows a couple’s relationship from their school years into later life and loss. It constructs nostalgia, love, and vulnerability through a synthetic flow of memories and dream-like spaces. Trillo (2024) has described the work as an exploration of memories that never occurred but nevertheless feel familiar (Pitchfork, 2024).

The video’s creator, director Paul Trillo, states that the seed of the idea occurred to him approximately 10 years ago and that he had waited all these years to realise it. He further explains that, to convey the video’s emotion, he deliberately used Sora’s dream logic and hallucinatory structure and focused on exploring memories that never occurred but feel as though they did (Trillo, 2024).

Romantic couple figures occupy the centre of the video’s emotional representation. The presence of a female–male couple identifies the relationship represented, but does not by itself demonstrate heteronormativity. Settings such as abstract spaces, a school environment, car scenes, and night journeys function as emotional thresholds associated with the couple’s shared past, their time together, and their loss. Fluid AI-generated transitions interrupt the linear structure of time and organise visual cues associated with dreaming, romance, and sorrow. In other words, the emotion in this story is constructed through cultural and spatial circulation. Abstract and uninterrupted transitions between spaces and events unfold fluidly without remaining bound to physical reality. The visual world created here functions not as a re-representation of reality, but rather as the synthetic production of memories that never occurred yet feel as though they did. Without laying claim to realistic physical consistency, this visual world offers imagery structured by memory and dream—in other words, a simulational reality. It illustrates how AI-assisted imagery can represent an emotionally meaningful world and accords with the idea, as formulated in Jean Baudrillard’s Theory of Simulation, that simulation ceases to be a copy representing reality and becomes a phenomenon that needs no reference beyond itself and produces its own reality. It may therefore be associated with debates on hyperreality and simulation.

Baudrillard (2010) account of simulation is useful here because it shifts attention from whether an image faithfully copies reality to how an image constructs a reality of its own. The transitions are not presented as though they belong to a continuous physical world. Memory, dream, and loss give these discontinuous sequences their affective significance.

In ‘The Hardest Part,’ spaces, bodies, and transitions do not follow the ordinary causal rules of the physical world. The interior of a car may open onto a different setting, while figures enter an abstract white space without a conventionally continuous passage between the two. Although the physical logic remains unstable, the synthetic world produced by these shifts is emotionally intelligible.

The stronger basis for a reading of heteronormative intimacy is the narrative organisation of the couple’s relationship. School encounters, journeys, and later-life change are gathered into a continuous account in which the female–male bond provides the recurring reference point for love and loss. At the descriptive level, these are scenes of a heterosexual romance; at the interpretive level, their organisation makes that romance the taken-for-granted framework through which an emotional life becomes intelligible. This supports a limited reading of conventional couple-centred intimacy. It does not establish an explicit rejection of other relationships, nor does it make every female–male image intrinsically heteronormative. The synthetic transitions destabilise physical continuity more clearly than they destabilise this familiar romantic framework.

Vulnerability in the video is constructed primarily through the temporal loss of the relationship and the transience of bodies. Rather than directly dramatising themes such as ageing, separation, and death, the AI aesthetic conveys them through the blurring of visual transitions and the unreality of the spaces. This shows that emotion is produced not only through bodily expression, but also through atmospheric and spatial coding.

This reading illustrates the movement from observation to interpretation. The school and journey settings, recurring couple, and discontinuous spatial transitions provide the descriptive material; emotional memory and couple-centred intimacy organise those observations at a broader level. PE3’s general emphasis on bodily, spatial, and editing cues helps explain why facial expressions alone would offer an incomplete reading. The connection is made here by the authors: PE3 did not view or comment on this video. IP3’s account of creating atmospheres beyond the constraints of a camera similarly provides a professional comparison, without establishing Trillo’s actual decisions.

This case suggests that generative AI can change the form of emotional narration as well as the production workflow. The nostalgia rests on a synthetic reconstruction of memory, not a record of an actual past. Within that reconstruction, the heterosexual couple continues to provide a familiar gendered account of love, loss, and vulnerability.

#### Militarised masculinity, anger, and threat: disturbed – bad man

In Disturbed’s ‘Bad Man’, images generated with Midjourney are assembled into a moving sequence with a visual rhythm reminiscent of stop motion. Accounts of its production indicate that around 10,000 generated frames were used and that assembly took roughly 1 month (LouderSound, 2022; Stash Media, 2022).

At first, political figures appear amid lightning; the video then turns to a conflict-ravaged dystopian landscape. The result is disturbing and immersive. Stark individual frames move in time with Disturbed’s aggressive musical arrangement, particularly its sharp guitar riffs and forceful drums. David Draiman, the band’s vocalist and songwriter, has explained that the song addresses the many “bad men,” or leaders, responsible for destruction around the world. Its immediate inspiration, however, was Russia’s invasion of Ukraine, which was taking place while the song was being written. Director Tristan Holmes likewise relates the work to the tragedy experienced by the Ukrainian people, describing it as a pointed response to the growth of autocracy in Eastern Europe and elsewhere (LouderSound, 2022; Stash Media, 2022).

No single historical figure is identified. Instead, uniforms, crowds, authoritarian faces, and ruined landscapes recur as a broader visual language of threat and power. The analysis accordingly attends to the emotional and gendered pattern that emerges across these images.

The impression of movement comes from placing generated still images in rapid succession rather than from conventional filming or animation. As a result, visual continuity gives way to a fragmented sequence driven by abrupt collisions between images. Midjourney’s painterly, highly stylised, and often surreal output reinforces this effect. Faces, bodily proportions, and spatial perspectives do not aspire to photorealism; together, they create a dark and symbolic dystopian atmosphere. This aesthetic is well suited to the video’s concern with authority, violence, corruption, and power. Historical and geographical references drawn from the song are used less to fix the action in one place or period than to give the threat a wider reach.

The stop–start quality interrupts seamless visual continuity and makes the succession of discrete images formally conspicuous. This can be interpreted as foregrounding the constructed image, but it does not establish whether viewers recognise the frames as AI-generated. The analysis concerns the organisation of the images rather than a measured state of audience awareness.

Stop motion creates movement from a sequence of incrementally different still images (Purves, 2010). Although the frames in ‘Bad Man’ were generated rather than photographed from physical objects, their rapid succession produces a related stop–start quality. Here that roughness becomes an expressive device rather than a technical defect.

The male body, soldier and authoritarian-leader figures, and scenes of mass anger establish a form of gender coding in the video. Masculinity is represented here through emotions and attributes such as power, control, authority, threat, and anger. These representations may be associated with a code that can be termed militarised masculinity. Flashes of lightning, a dark atmosphere, harsh and angry facial expressions, and dystopian settings connect the representation of masculinity with anger and fear.

From the perspective of emotional representation, these representations of threat and anger are produced through the visual rhythm of the stop-motion-like assembly of generated images. Instead of fluid cinematic movements and transitions, trembling, staccato, and collision-driven images give the sequence an aggressive formal character. Its discontinuity interrupts the expectation of smooth movement. Moreover, the rough, imperfect, and non-fluid flow of the visual world combines with the song’s hard rhythm and aggressive vocals to intensify a sense of loss of control, aggression, and impending danger.

The concentration of authority, war, and threat in male bodies is also relevant to debates on representational bias. The video recirculates a familiar association between masculinity and power, even while presenting that association within a critical, dystopian narrative.

The video should not, however, be read as endorsing these codes. Rather than glorifying representations of aggressive, authoritarian, and militarised masculinity, its dystopian and dark atmosphere and images of aggressive faces associate them with threat and corruption. The video therefore demonstrates that generative-AI visuality can both reproduce familiar stereotypes and render them visible within a critical aesthetic. The capacity to discuss these two movements together is decisive for the analysis.

#### AI-assisted femininity, desire, and female anger: Björk ft. Rosalía – Oral

Carlota Guerrero’s video for Björk and Rosalía (2023) song “Oral” uses a hybrid production process. The official credits identify a director of photography, choreographer, stunt coordinators and doubles, an editor, and a VFX team (Björk and Rosalía, 2023). The credits establish the contribution of filmed performance and production labour. The release account, meanwhile, identifies AI-assisted likenesses and glitches intended to question identity and the distinction between reality and virtuality (Pitchfork, 2023). Neither source warrants describing the bodies, choreography, or setting as wholly generated. We therefore consider the altered likenesses and avatar-like appearance separately from the performed movements and other production choices. The account also describes the combat as preparation for a shared threat, which gives the apparent confrontation a context of solidarity.

Figures bearing the two artists’ likenesses are shown wielding swords and performing combat and defensive movements in a surreal white setting. Rather than a conventional plot, the video follows a flow built around themes and events such as two bodies confronting and touching one another, falling, and rising again. Although a first viewing may suggest a conflict between two women, the production account identifies the characters as allies preparing against a greater common threat (Pitchfork, 2023). Thus, instead of the theme of rivalry between women familiar from popular culture, the video presents solidarity and collective action.

Anger, emphasised in the production account, is visualised not through emotions such as uncontrolled power or jealousy, but as a disciplined and resolute energy. Ahmed (2014) argues that emotions are not confined to an individual’s psychological state; they also work as cultural and political practices through which communities are formed. Here, anger binds the women to a common struggle; it does not divide them into rivals.

The female figures perform acts of strength, combat, threat, and attack commonly associated with masculinity. They remain culturally recognisable as women through their faces, hair, and bodily form. Against the white setting and plain costumes, attention falls on their positions and movements. Femininity is retained, but it is associated with agility, anger, and physical power rather than passivity and vulnerability. This differs from a pattern reported in research on generative AI, in which women tend to appear younger, more often smiling, and happier, while men appear older and angrier (Zhou et al., 2024). The women in “Oral” have physical agency and can express anger. Their actions are, however, choreographed and visually polished. Performance, direction, and editing must be considered alongside AI in accounting for that representation.

The artists’ likenesses make the figures recognisable even as visible alterations complicate their relation to the performers. Smooth surfaces and the carefully composed setting aestheticise femininity. To describe this as eroticisation would require further evidence: sexualised framing, fragmentation of the body, or a gaze that makes display more important than action. The combat and defensive movements instead give the figures practical agency. The reading supported here is that bodily strength and solidarity accompany a polished feminine appearance. Casting, costume, lighting, selection, AI-assisted alteration, and post-production may all contribute to that appearance.

The codes distinguish what these features support. Movement in combat provides evidence of agency and controlled aggression; smooth appearance supports aestheticisation. Neither identifies a model’s separate contribution. IP1’s account of selecting and revising outputs qualifies a purely algorithmic explanation, while IP6’s emphasis on consent helps distinguish the official use of an artist’s likeness from an unauthorised imitation. These are connections made by the authors between general professional accounts and the case, not statements by either participant about working on “Oral.”

In “Oral,” anger is neither medicalised nor reduced to personal pathology; it becomes political. Competition between women gives way to solidarity, while the female body shifts from an object of spectatorship to a source of action. The combination of recorded performance and altered likenesses complicates the boundary between the real and the artificial. Against the stereotype of the emotional, passive, and submissive woman, the video produces an alternative representation of femininity through collective resistance and elements such as power, anger, and attack. The polished treatment of the figures and the aestheticisation of anger, however, mean that a departure from passive femininity does not necessarily displace familiar appearance norms. The video’s critical value arises not from resolving this contradiction, but from visibly destabilising the boundaries among the female body, technological representation, subject, and avatar.

#### Bodily ambiguity, gendered narrative, and synthetic emotion: Peter Gabriel – Panopticom

The Lamson Official Video produced for Peter Gabriel – Panopticom (Dark-Side Mix) is one of the AI-assisted music videos created and officially released as part of the #DiffuseTogether competition organised in collaboration between Peter Gabriel and Stability AI. The competition reportedly invited participants to create animations inspired by Peter Gabriel’s songs using Stability AI technologies, and the Lamson version was among the winning videos selected in this context (Gabriel, 2023a,b; Stability AI, 2023; PM Studio, 2023).

Panopticom combines uncertainty in the appearance of some synthetic bodies with a recognisably gendered narrative. The movement away from a stable human form should therefore be distinguished from the roles assigned to characters. Within the video, cyborgs and robotic figures share a visual environment with surveillance devices, organic tissue, and mechanical surfaces. Conventional signs by which a human body is gendered, including face, skin, muscle, and hair, are partly displaced by metal, cables, lenses, and components resembling prostheses. At the level of bodily appearance, these changes can make familiar gender cues less legible. They do not, on their own, establish that gender ceases to organise the story.

The video’s principal emotional atmosphere is shaped around paranoia, the sense of being watched, anxiety, and alienation. The relationship between surveillance and emotion occupies the centre of the work. The ambiguity of human expressions on robotic bodies with unstable human features provides cues that can be read in terms of curiosity or discomfort. Recurrent eyes and surveillance imagery and dystopian settings construct a sense of being under constant observation. The eye represents not merely an instrument of vision, but also the power to classify and control. This visual order may be associated with Foucault’s (1977) concept of the panopticon. In the panoptic order, powers use the techniques of the gaze that they develop to control and discipline individuals and their bodies. Even if individuals do not know whether they are actually being watched, they regulate their own behaviour in view of the fact that they may be watched at any moment (Tümurtürkan, 2010).

Human bodies are not presented in a stable and integrated form in this video. Instead, the figures transform into skeletons, technological avatars, animals, abstract entities, and unconventional forms. Fluid transitions blur and destabilise the body’s boundaries and present human identity as a constantly changing structure. Before a stable figure can be recognised, the body or character in the image transforms into a different form, destabilising the distinctions between human and machine. Transitions among trees, plants, skeletons, bodies, and scenes unfold within a cyclical relationship between life, death, and rebirth. These transformations distribute emotional meaning across setting, colour, bodily change, and technological forms rather than confining it to facial expression. Their meaning also depends on the narrative in which they occur.

In Lamson’s account, a peaceful man is drawn into a surveillance system and becomes a cyborg. Feelings for a female cyborg restore his capacity for emotion. He eventually gives her his heart, allowing her to return to human form (Gabriel, 2023b). The changing bodily surfaces therefore belong to a story with gendered positions: the man desires and sacrifices, and the woman receives the gift that restores her. A director’s explanation cannot settle every interpretation of a work, but this account makes a claim of gender neutrality difficult to sustain. Bodily ambiguity remains part of the imagery while male and female roles continue to structure desire and sacrifice.

This reading changes the comparison with The Hardest Part. Both works give a male–female relationship an organising role, despite their different bodies and settings. In Panopticom, transformations disturb human appearance without displacing the familiar allocation of desire and agency within the romance. PE2’s general comments on the difficulty of naming emotion in non-human faces offer a useful interpretive question at the level of appearance. They do not establish the characters’ gender or override the narrative account. An initially broad code of ambiguity is thus narrowed to the bodily level and considered alongside narrative gendering.

#### Interviews with industry representatives: AI, emotion, gender, and creative responsibility in the production chain

This section was structured to relate the themes emerging from the visual analyses to the experiences and views of workers in the sector. The data obtained through the interviews were considered within the article’s analytical framework, and both direct quotations and summarised statements are presented.

Asynchronous written interviews were conducted with six industry representatives involved at different stages of AI-assisted music-video and visual production. They are coded as IP1–IP6. Their experience across idea development, prompt writing, creative production, editing, and publication provided a general production-oriented perspective that the authors related to the four cases. None had worked on these productions.

All six industry interviews were included in the coding and cross-participant thematic synthesis. IP2 and IP4 are not reproduced as stand-alone verbatim quotations in the excerpts below; their responses were nevertheless included in the analysis. Quotations were chosen when they conveyed a theme clearly or offered a distinct way of expressing it. Where accounts overlapped, the shared material was presented in thematic summaries. References to “participants generally” consequently concern the full set of interviews, including IP2 and IP4.

The first dimension of the interview analysis concerned AI’s place in production. Across the responses, participants described its influence on aesthetic choices and production decisions as well as its capacity to speed up technical work.

*“Although for some time I used artificial intelligence primarily for preparation, pre-production, or creating storyboards, I have recently also begun to use it to construct an atmosphere that could not be created with a camera, to create scenes that would be impossible to film, and to produce visual effects. I can say that it broadens my creativity and horizons.” (IP3, director)*

The authors relate this general account to the surreal universe in Washed Out – The Hardest Part. In that video, artificial intelligence is not merely a tool that facilitates or accelerates production; rather, it is used to create surreal settings, shots, and transitions within a fluid timeline organised around themes such as reality, dreams, and nostalgia, producing images that would involve different constraints in a conventional workflow. Similarly, Peter Gabriel’s Panopticom contains surreal sequences in which the idea of an abstract technological gaze is constructed through non-human entities and abstract visuals. This comparison suggests how AI-assisted production can enter the construction of atmosphere. IP3’s account does not, however, verify the workflow of either selected video.

One interview theme concerned the language used for emotion and gender in prompts. Participants generally described prompts not as purely technical commands, but as written directions through which cultural codes enter the production process.

*“I describe the emotions/expressions I want to reach or obtain clearly, as though I were speaking to a person. When expressions such as ‘melancholic woman,’ ‘powerful woman,’ ‘threatening man,’ or ‘authoritarian leader’ are used in a prompt, the model immediately moves towards certain patterns. Female figures may emerge with younger, smoother, and more vulnerable faces; male figures may have harsher gazes, darker colours, and costumes suggestive of the military or of power.” (IP5, director)*

The account describes IP5’s experience of an interaction between prompt wording and recurring outputs; it does not isolate the effect of either factor. From this perspective, prompting is also a cultural practice: it is one point at which emotional and gendered codes may be inserted, reproduced, or intentionally challenged.

For IP1, filtering the generated material mattered as much as producing it. Negative prompts, reference images, repeated generation, and manual revision influenced which representations were retained in the finished work.

*“If you accept the model’s first output, you generally obtain an average, safe, and clichéd aesthetic. If you want more diverse bodies, different ages, different gender expressions, or less stereotypical emotions, you have to ask for them explicitly. In other words, diversity does not emerge on its own; it must be deliberately incorporated into the prompt and the selection process.” (IP1, art director)*

This response highlights the need to approach generated outputs critically. In IP1’s account, diversity, stereotypes, gender roles, and emotional codes depend on what the producer requests, selects, rejects, and edits as well as on the generated material. This qualifies the attribution of polished or stereotyped appearances in the visual analyses to an algorithm alone.

Another issue that must be addressed in relation to the subject is creative responsibility. In this dimension, matters such as copyright, deepfakes, consent, diversity, and transparency in AI-generated music videos should be regarded as processes that create ethical responsibilities not only during production, but also at the stages of publication and circulation. It is known that debates over copyright and ownership in works produced with AI assistance can have adverse effects on artists’ emotional states and their desire to create (Kambur and Dolunay, 2024).

*“The use of an artist’s own avatar with their consent and the imitation of their face, body, or voice without permission are very different situations. Music videos are far more than visual outputs; they are among the most important components of an artist’s image. For this reason, issues such as consent, copyright, and image in the use of artificial intelligence must be carefully evaluated from the perspectives of both the producer and the artist.” (IP6, artist)*

Björk ft. Rosalía’s ‘Oral’ provides a useful example, as its synthetic likenesses are part of an official production involving the artists. Using a likeness with authorisation raises different ethical questions from reproducing someone’s face, body, or voice without permission. Consent, context, and control remain central to that distinction.

IP3 also drew attention to the responsibility to disclose the use of artificial intelligence to audiences:

*“When content generated with artificial intelligence is presented to the public, it should be transparently and explicitly identified as having been generated with AI, and such content should be labelled. Otherwise, in an uninformed and non-transparent structure/presentation, the public may become unable to distinguish what is real from what is fake; this would not only be unethical, but could also lead to more serious problems.” (IP3, director)*

The participant’s concern extends ethical responsibility beyond production to the communication established with audiences. AI-generated visuals may strengthen an artist’s image and expand a work’s aesthetic range, but they may also blur the boundary between recorded and synthetic material. Clear disclosure can help audiences interpret that boundary.

An overall evaluation of the interviews shows that representations of gender and emotion in AI-assisted music videos are shaped at three principal stages. The first is prompt writing. At this stage, the desired emotional and gender codes are communicated to the generative system through the prompt. The second is algorithmic production. At this stage, the generative model produces a range of possible outputs that may carry familiar codes from visual culture. The third is the stage of filtering, post-production, and decision-making. Working from the images generated by AI, the human producer uses negative prompts and revisions to intervene manually in the areas they wish to change and obtain the final output, choosing how particular stereotypes will be modified and deciding which visuals will be used. Because the study did not audit training data or recover complete production logs for every case, these stages are treated as interdependent rather than as isolated model effects.

AI-assisted music videos must therefore be discussed at the level of production decisions as well as visible outputs.

Gender representations and emotional codes in AI-assisted music videos do not consist solely of the outputs visible on screen. Rather, they take shape across several connected stages: prompt writing, algorithmic generation, selection, post-production, decisions concerning production and the artist’s image, ethical review, publication, and circulation. Generative AI in music-video production should therefore be evaluated not only through aesthetics or convenience, but also in terms of emotional meaning, ethical values, and professional responsibility across the production chain.

#### Interviews with psychology experts: emotion recognition, body image, and the synthetic visual experience

The psychology experts responded to general questions about emotion, gender, and synthetic imagery, without viewing the four videos or receiving the authors’ preliminary interpretations. Their responses are organised here around emotion recognition, synthetic bodies, representation, and ethical concerns. The case connections below are analytical connections made by the authors, rather than case-specific expert assessments or evidence of audience effects.

PE3 argued generally that emotional representation in AI-assisted imagery should be read not only through facial expression but also through the body, colour palette, editing, positioning, and movement.

*“If emotion is sought only in the face in videos of this kind, much will be missed. Because facial expressions can be excessively perfect in AI-generated videos, basic emotions may not appear natural. In such cases, details such as the body, colour palette, editing, bodily positioning, and movement become more decisive in conveying emotion.”*

The authors relate this general observation to The Hardest Part and Panopticom. In the former, spatial transitions and the passage of time provide cues for nostalgia and vulnerability; in the latter, mechanical eyes, cyborg forms, and bodily change contribute to the representation of surveillance and alienation. PE3’s account supports attending to such contextual cues rather than relying exclusively on faces. It does not confirm these particular emotional labels, since the expert did not assess either video. The narrative qualification identified in Panopticom further shows why attention to visual atmosphere must be combined with attention to characters’ roles.

PE2 suggested that the absence of familiar facial expressions in non-human figures may make an emotion harder to name while contributing to atmospheres of alienation, curiosity, mystery, uncanniness, or distrust. Read alongside the visual findings, PE2’s emphasis on uncertainty qualifies a simple correspondence between a visual cue and a named emotion, while PE3’s account supports attention to context. Their responses therefore help identify both the value and the limits of the authors’ emotional interpretations.

PE4 and PE2 discussed body image, idealisation, and trust. PE4 was particularly concerned that repeated encounters with idealised synthetic bodies could invite comparison, noting that “young people in particular may overlook the fact that these images are artificial and compare themselves with them.” PE2 likewise linked flawless synthetic bodies to the normalisation of unrealistic standards and possible dissatisfaction. These are concerns raised by the experts; effects on viewers were not tested in this study.

PE1 emphasised transparency and social sensitivity when discussing ethical responsibilities, particularly for material that children and young people might encounter. This included clearly identifying AI-assisted content and avoiding the repeated promotion of unrealistic beauty standards.

## Discussion

For emotion science, the cases show why a face alone is an insufficient basis for reading emotional representation. We interpreted nostalgia, threat, desire, and uncanniness through movement, colour, setting, editing, and bodily form, with the music providing context. Synthetic aesthetics changed how these features were combined. The result is an account of how visual cues acquire culturally recognisable emotional meaning, not a measurement of inner states or a comprehensive analysis of sound–image relations. Across the videos, generated and altered bodies, spaces, and transitions do much of the work that a reading centred on facial expression would miss.

The videos also show that AI’s contribution extends beyond making production faster, cheaper, or technically easier. Each work contributes to a representational culture in which emotion is organised visually, bodies acquire gendered meanings, and synthetic images can still present viewers with convincing emotional worlds. These findings are consistent with the idea, accepted in the literature, that AI is not merely a tool in cultural production, but a socio-technical system shaped by user practices (Erbay and Joyce, 2025).

When the 4 cases selected for the study are considered together, it becomes apparent that emotions in music videos are treated not simply as fixed categories, but as active and variable clusters of representation within audiovisual arrangements.

While the video Washed Out – The Hardest Part produces emotions such as nostalgia, love, romance, and vulnerability through romantic memory, Disturbed – Bad Man presents emotional codes such as anger, fear, threat, and aggression through dense images of militarised masculinity. In Björk ft. Rosalía – Oral, female figures with AI-assisted likenesses combine emotional codes such as anger, desire, and threat with choreography and cooperation, offering a new perspective on female aesthetics; Peter Gabriel – Panopticom, in turn, combines surveillance anxiety and technological uncanniness with a gendered story of desire and sacrifice. Its cyborg forms complicate bodily appearance without eliminating gendered narrative roles.

Across the cases, particular emotional codes were connected with bodies, movements, and spaces that carry gendered meanings. The pattern is neither uniform nor attributable to AI alone.

In Washed Out – The Hardest Part, the temporal journey of a romantic couple is treated through themes of emotion such as nostalgia, loss, and romance. In this case, emotion is produced not directly through facial expressions, gestures, and movements, but primarily through atmosphere, space, transitions, and settings. The romantic couple’s memories of the past and emotional memory are associated with a conventional couple-centred framework of intimacy, interpreted here as a limited form of heteronormative organisation. In this respect, the case shows that love, romance, and vulnerability are represented within a synthetically reproduced field of memory. In Disturbed – Bad Man, by contrast, images such as militarised masculinity and authoritarian male bodies are associated with emotional codes such as anger, threat, and power. Considered together, the two cases show that different clusters of emotion are connected with different gender codes.

Björk ft. Rosalía – Oral represents female figures as active participants in training and combat, connecting femininity with strength, anger, and solidarity. The figures retain a polished appearance while exercising that agency. This supports a distinction between aestheticisation and passivity, rather than an assumption that idealised appearance necessarily entails eroticisation. It also requires caution about causal attribution: the documented hybrid production includes filmed performance, choreography, casting, styling, editing, and visual effects. From the finished images alone, it is not possible to identify the decisive contribution of any one process, including AI-assisted alteration.

In Panopticom, the distinction concerns how a body appears and the role it occupies in the story. Mechanical forms can make gender cues uncertain at the level of an individual image, while the story still organises desire and sacrifice through a male subject and female recipient (Gabriel, 2023b). It therefore qualifies any cross-case inference that non-human or mutable bodies necessarily move beyond familiar gender relations. Its contrast with The Hardest Part concerns the treatment of bodies and environments more clearly than the absence of a gendered romantic structure.

A central issue is the recirculation of patterns already familiar from media culture. Prior audits show that image-generation systems can reproduce gendered associations in occupations, facial expression, and personality traits (Naik and Nushi, 2023; Sun et al., 2024). Idealised femininity is also present in the cases considered here, alongside links between masculinity, authority, and threat. These patterns cannot, however, be attributed to the model in isolation, because the study neither examined its training data nor compared prompts systematically. Recent experimental work likewise found poorer body-image outcomes after exposure to idealised gen-AI fitness imagery than after exposure to body-diverse, functionality-focused imagery (Alleva et al., 2026).

AI-assisted production nevertheless accommodates fluid settings, changeable bodies, visually ambiguous figures, and non-human figures capable of conveying emotion. Producing comparable forms through a conventional workflow would involve different resource demands. Avatars and cyborgs can make established bodily categories less stable, although the narrative may still assign conventional gendered roles. Generative AI carries both representational risk and experimental potential; how that potential is used depends on creative and ethical decisions across the production process.

From the perspectives of psychology and affective science, the analysis concerns culturally constructed representations of emotion, rather than establishing the nature of internal emotional states. In the cases examined, emotional codes were not constructed solely through the characters’ faces and expressions; they were also supported through setting, colour palette, bodily images, editing, and synthetic aesthetics, and were at times used as the principal coding element. Music videos produced with the support of generative AI therefore offer a productive field of research for observing how emotions are connected with gender patterns.

The written industry interviews add a general perspective on production practice to the study. While the video analyses help interpret the forms of representation visible on screen, the industry interviews identify professional decisions that complicate the attribution of an observed representation to the model alone. Because participants did not work on the cases, these accounts cannot establish their specific production histories. The findings suggest that it is inappropriate to assign ethical and representational responsibility to generative AI models alone when using such systems; human decisions and professional responsibility play an important role at every stage of the process. Dolunay and Temel (2024) examine unethical AI use in academia rather than creative production. Their relevance here is limited to the broader issue of responsibility for human choices surrounding AI use; their findings are not evidence about music-video workflows.

The general psychology-expert responses contribute a further interpretive perspective. PE3’s attention to bodily and contextual cues supports the breadth of the visual framework, while PE2’s emphasis on uncertainty cautions against treating particular cues as unambiguous indicators of emotion. PE4’s concerns about idealisation raise questions for future reception research rather than demonstrating an effect of the selected videos. Integration therefore rests on specifying what each source adds and where its explanatory reach ends. The experts’ responses neither validate individual case readings nor establish what audiences felt.

### Ethical and practical implications

It must not be overlooked that, in cultural and creative fields, the public may fail to recognise AI-assisted productions as outputs generated by artificial intelligence. In such cases, the use of AI must be disclosed clearly in order to follow the most appropriate course in terms of representational responsibility and ethics (Haraç and Dolunay, 2026). Producers working in this field should be able to recognise recurring patterns of gender, body, and emotion in outputs obtained from AI systems and should make deliberate choices concerning diversity and sensitivity. Prompt writing in the production process should be seen not merely as a tool for generating visuals from text, but also as an ethical stage at which decisions about representation are made. The outputs obtained from those prompts also determine the stereotypes, emotions, spaces, and codes with which the synthetic visual world to be created will be associated.

The practical recommendations highlighted within the study are as follows: rather than being finalised solely on the basis of aesthetic concerns, visuals generated through prompts should also be evaluated in terms of their representational effects; diversity in factors such as gender representations, gender ambiguity, age, body type, and character form should be monitored; and, finally, the role of generative AI in the process of cultural production should be disclosed transparently to viewers.

These proposals are intended to support ethically, culturally, and socially responsible production, rather than to limit the use of generative AI. Through their wide circulation, music videos give social assumptions about bodies, identity, desire, and adequacy a visible form. Those assumptions may be reinforced, but they may also become open to criticism. AI outputs therefore entail responsibilities for the production team not only at the technical level, but also in terms of oversight of representation.

The written responses from psychology experts add another perspective to the project. The principal idea drawn from these interviews is that creative teams should evaluate not only the social meaning and technical aspects of their representations, but also their possible psychological implications for viewers. Factors such as idealised bodies, perfected faces, images of authoritarian masculinity, the consensual or non-consensual use of avatars, and the sense of uncanniness that cyborg bodies may create should be considered separately at the publication and distribution stages. AI productions should therefore be addressed and reviewed in terms of psychological impact and representation alongside technical controls, and the review stage should be evaluated with an awareness of ethical responsibility. These implications were raised by the experts rather than measured directly in this study.

### Limitations and future research

This qualitative study is based on a purposive, theory-driven sample of four music videos and 10 expert respondents, so its findings are not statistically generalisable. Two of the eight invited industry professionals and two of the six invited psychology experts did not respond; the achieved sample may consequently reflect willingness and availability to participate. The single-round written procedure allowed considered responses but limited follow-up probing and provided no non-verbal interview cues. Turkish responses were translated by the authors, and the English quotations should be read with the possibility of linguistic nuance in mind. Coding was undertaken jointly, without an independent recoding exercise. Psychology experts were not shown the videos, and industry participants had not worked on them, so neither source independently verifies the case interpretations or production histories. Music and lyrics were used to contextualise the images and were not coded as a separate corpus. We had no audience testing, non-AI comparison group, model training data, or complete production records. The material thus offers no way to separate model effects from performance, prompting, selection, direction, editing, and other creative choices. Further work could ask experts and audiences to respond to specific videos, compare AI-assisted and non-AI productions across genres, or examine complete production records.

## Conclusion

The four videos show how synthetic visual culture gives form to love, nostalgia, power, threat, desire, and anxiety. Generative systems take part in this process through the bodies and settings they help produce. Emotional meaning emerges from atmosphere, colour, movement, editing, faces, and bodily form, with gender conventions sometimes retained and sometimes questioned.

Neither the persistence nor the removal of stereotypes describes the sample adequately. Heteronormative intimacy, militarised masculinity, and idealised femininity appear alongside female anger, solidarity, and changing synthetic bodies. Panopticom makes the tension particularly clear: an ambiguous bodily appearance can belong to a conventionally gendered story. The works can bring such contradictions into view without resolving them.

These readings connect research on generative AI to questions of gender and emotional representation in synthetic visual culture. Familiar representational patterns recur, but the videos can also make those patterns available for scrutiny. AI-assisted production consequently calls for attention to bias and gender conventions, while offering artists ways to experiment with identity, bodies, and emotional expression.

The theoretical contribution is an approach to AI-assisted music videos as synthetic environments in which emotional codes and gendered meanings are organised. This directs attention beyond technical novelty to the relations among movement, faces, bodies, and the cultural coding of emotion. The interpretive framework brings those relations into a shared discussion across affective science, visual culture, media and communication, gender research, and AI ethics.

The psychology experts helped us question the readings by drawing attention to context, idealisation, and uncertainty. Their general responses cannot establish what audiences make of the four videos. Read alongside the industry accounts, they contribute professional perspectives to the interpretation while keeping visible representation, production experience, and possible reception distinct.

For creative practice, the findings point to a need for review at each stage, from writing prompts and selecting images to publishing and circulating a video. Technical quality, cost, and speed are relevant, but so are the emotional meanings and gender representations being put into circulation. Attention to these choices, and to the responsibilities of those making them, can support more pluralistic and ethically considered production.

## Data Availability

The original contributions presented in the study are included in the article/supplementary material, further inquiries can be directed to the corresponding author.
